# Correlation between galectin-3, RDW, Hepc, HS and ferritin and prognosis of patients with acute onset of chronic heart failure

**DOI:** 10.1186/s12872-022-02847-8

**Published:** 2022-11-08

**Authors:** Lingling Yao, Yanjie Tan, Fei Chen

**Affiliations:** Department of Cardiology, Renmin Hospital, Hubei University of Medicine, No. 39 Chao Yang Road, Shiyan, 442000 Hubei China

**Keywords:** Galectin-3, RDW, Hepc, HS, Ferritin, CHF acute attack, Prognosis, Correlation

## Abstract

**Background:**

Chronic heart failure (CHF) is characterized by a high hospitalization rate and a high mortality rate. It is particularly important to identify biomarkers for predicting the prognosis of patients with acute attack of CHF.

**Purpose:**

To observe the correlation between galectin-3, RDW, Hepc, HS and ferritin and the prognosis of patients with acute onset of CHF.

**Methods:**

The study included 92 patients with acute onset of CHF who received treatment at our hospital between August 2020 and December 2021. After treatment, the patients were divided into the effective group and the non-effective group based on the effectiveness of treatment. The levels of galectin-3, RDW, Hepc, HS and ferritin before and after treatment were compared between the two groups and the correlation between prognosis of patients with acute attack of CHF and galectin-3, RDW, Hepc, HS and ferritin was observed.

**Results:**

The effective rate was 71.74% (66/92) and the ineffective rate was 28.26% (26/92) in the 92 patients with acute attack of CHF in the study. Before and after treatment, the levels of galectin-3, RDW, Hepc, and HS were lower in the effective group than those of the non-effective group while the level of ferritin was higher in the effective group than that of the non-effective group (*P* < 0.05). Spearman correlation analysis showed that the level of prognosis of patients with acute attack of CHF was positively correlated with galectin-3, RDW, Hepc, and HS (r = 0.217, 0.109, 0.376, 0.765, P = 0.026, 0.032, 0.021, 0.006), and negatively correlated with ferritin (r = − 0.127, *P* = 0.037). The independent variables were galectin-3, RDW, Hepc, HS and ferritin and the dependent variable was prognosis of patients with acute attack of CHF. Univariate logistic regression analysis showed that alectin-3, RDW, Hepc, HS, and ferritin were protective factors for the prognosis of patients with acute attack of CHF. The independent variables were galectin-3, RDW, Hepc, HS and ferritin, dependent variables and the dependent variable was prognosis of patients with acute attack of CHF. Multivariate logistic regression analysis revealed that galectin-3, RDW, and Hepc were risk factors of the prognosis of patients with acute attack of CHF.

**Conclusion:**

Galectin-3, RDW, Hepc, HS and ferritin were closely related with the prognosis of patients with acute attack of CHF and galectin-3, RDW, and Hepc were risk factors of the prognosis of patients with acute attack of CHF.

## Background

Chronic heart failure (CHF) is a complicated multifactorial clinical syndrome. The incidence of HF in adults in foreign countries is about 1–2% and exceeds 10% in persons aged above 70 years. Its 5-year mortality rate is > 40% and the survival rate is far lower than that of most cancer patients [1]. CHF is characterized by a high hospitalization rate and a high mortality rate. Acute attack of CHF can be relieved by conventional treatment, but some patients have a poor prognosis [2].

In clinical practice, it is particularly important to identify biomarkers for predicting the prognosis of patients with acute attack of CHF, which is critical for early prevention and treatment of acute attack of CHF. B-type natriuretic peptide (BNP) and N-terminal pro-BNP (NT-pro-BNP), are important biomarkers for heart failure screening, diagnosis and differential diagnosis, assessment of disease severity and prognosis in the clinical practice, but their levels and efficacy are affected by age, the renal function, cardiovascular and non-cardiovascular diseases. Therefore, it needs to find other molecules to act as biomarkers for heart failure. Recently, hepcidin (Hepc), ferritin, galectin-3, red cell distribution width (RDW), and heparan sulfate (HS) show a possibility to act as biomarkers for heart failure. Iron deficiency may trigger the symptoms of heart failure, leading to a poor prognosis [3]. Hepc is a major regulator of iron metabolism, and may be associated with CHF [4]. Ferritin is a complex of apoferritin and iron (Fe3+) core, and is mainly involved in hematopoiesis and regulation of the immune system, and is an important iron storage protein and also a key protein in the regulation of iron metabolism [5]. CHF patients may have serum ferritin abnormalities, which may be associated with the onset and development of CHF [5]. Galectin-3 a biomarker of cardiovascular disease and has drawn attention recently; it is involved in ventricular remodeling and regulation of interstitial fibrosis [6]. RDW is a parameter that reflects the heterogeneity of the volume of peripheral blood red blood cells and may be associated with prognosis of heart failure [7]. HS is a marker of glycocalyx on the surface of vascular endothelial cells and is involved in inflammatory reaction [8]. Therefore, it is hypothesized that serum galectin-3, RDW, Hepc, HS and ferritin are associated with the prognosis of patients with acute attack of CHF. However, few reports are currently available. Therefore, the current study explored the correlation between galectin-3, RDW, Hepc, HS and ferritin and prognosis of patients with acute attack of CHF.

## Method

### Subjects

The study included 92 patients with acute onset of CHF who received treatment at our hospital between August 2020 and December 2021. Their age ranged from 50 to 79 years, with a mean age of (59.67 ± 11.52) years. There were 45 male patients and 47 female patients. The NYHA cardiac function at admission was class II in 23 cases, class III in 26 cases and class IV in 43 cases. 20 patients had hyperlipidemia, 19 had diabetes, and 27 had hypertension. 17 patients were smokers and 12 were drinkers. The inclusion criteria includes the diagnostic criteria of CHF based on China Guidelines on the Diagnosis and Treatment of Heart Failure (2018) [9]; in the acute exacerbation stage of CHF, there are obvious signs of water and sodium retention and systemic circulation congestion; clear duration of acute attack of CHF; good compliance, and mentally normal. Exclusion criteria: acute coronary artery syndrome or coronary artery reconstruction or other major surgeries within the past 3 months; anemia or iron supplementation therapy for the past 12 months; any acute or chronic disease, diseases that may influence iron metabolism including malignancies, severe kidney disease requiring hemodialysis and hematological diseases; severe hepatic and renal insufficiency; autoimmune disease; acute cerebral infarction, septic shock, atrial fibrillation and malignant tumors that may influence the levels of serum chemokines and HS; pregnant or lactating women.

### Treatment and evaluation of prognosis of patients with acute attack of CHF

(1) Treatment: All patients received standard of treatment based on patient's condition. The drugs were as follows: hydrochlorothiazide tablets (#H32021683, Changzhou Pharmaceutical Factory Co., Ltd., China), 25 ~ 50 mg, 1 ~ 2 times / day; perindopril tablets (#H20020226, Les Laboratoires Servier Division Oril, France), 4 mg/day, taken once in the morning; metoprololcan be intravenously injected at 2.5–5 mg / time (2 min), once every 5 min, a total of 3 times of 10 ~ 15 mg. After 15 min, start to take 25–50 mg of metoprolol tartrate tablets (#H32025391, AstraZeneca Pharmaceuticals Co., Ltd., USA) orally, once every 6–12 h, for a total of 24–48 h, and then take orally 50–100 mg/time, twice a day; digoxin tablets (#H33021738, Sanofi Pharmaceuticals Co., Ltd., Hangzhou, China); irbesartan tablets (#J20130049, Sanofi Pharmaceuticals Co., Ltd., Hangzhou, China), 150 mg / time, once a day; levosimendan (#H20100043, Qilu Pharmaceutical Co., Ltd., China), slow intravenous injection within 6–12 μg/kg·10 min, and then the dose was adjusted to 0.1 μg/(kg·min), and the patient’s tolerance was observed after 1 h. If the patient tolerated well, the dose was adjusted to 0.2 μg/(kg·min), and the drug was maintained for 24 h and repeated 1 time after 7 days; ferric sucrose (#H20046043, Nanjing Hengsheng Pharmaceutical Co., Ltd., China), 5 ml, intravenously twice a week, and stopped until the level of ferritin recovers to 30–200 μg/L.

(2) Evaluation of Clinical efficacy: Treatment efficacy was evaluated after 48 h. dyspnea and shortness of breath were alleviated; there was no dyspnea at rest after lying down in the supine position for 24 h; rales disappeared in the lungs; serum NT-proBNP levels were reduced to less than 30% of that at admission; the heart rate was 10%-15% lower than that at admission. The 92 patients with acute attack of CHF were divided into the effective group and the non-effective group according to treatment efficacy.

(3) Collection of clinical data: Clinical data including age, sex, NYHA cardiac function class, comorbidities (obesity, COPD history, hyperlipidemia, diabetes, hypertension), history of smoking and drinking, the data of B-type natriuretic peptide (BNP), N-terminal pro-BNP (NT-pro-BNP), and cardiac function (Philips EPIQ-7 color Doppler ultrasound detector, USA) were collected from all the study subjects.

(4) Galectin-3, RDW, Hepc, HS and ferritin: Ten mL fasting peripheral venous blood was obtained at admission and 2 days after treatment. Half the sample was analyzed for RDW using an automatic blood cell analyzer and the half of the sample was left at the room temperature for 1 h and centrifuged for 15 min at 3000 r/min with a centrifugal radius of 10 cm and the supernatant was saved. Hepc, HS, ferritin, and galectin-3 were measured by ELISA using commercially available kits (RD, USA) as instructed by the manufacturer and the results were read using an ELx800 microplate reader (BioTek, USA).

(5) Follow-up: the occurrence of readmission due to CHF, cardiac death, malignant arrhythmia, and acute coronary syndrome within 6 months was recorded.

### Observation parameters

The levels of galectin-3, RDW, Hepc, HS and ferritin before and after treatment were compared between the two groups. The correlation between the prognosis of patients with acute attack of CHF and galectin-3, RDW, Hepc, HS and ferritin was observed. Univariate/multivariate logistic regression analysis was carried out to identify the risk factors of the prognosis of paitens with acute attack of CHF.

### Statistical analysis

Data were analyzed using SPSS26.0 software. Normally distributed data were expressed in $\overline{\user2{x}}$ ± s and comparison between two groups was done using independent sample t test between two groups. Non-normally distributed data were expressed in median (quartiles) [M (Q_1_, Q_3_)] and comparison between two groups was done using Mann–Whitney U test. Intragroup comparison of different time points was done using Wilcoxon rank sum test. Categorical data were compared using χ^2^ test. Spearman correlation analysis was done to investigate the correlation between the levels of galectin-3, RDW, Hepc, HS and ferritin at admission and the prognosis of patients with acute attack of CHF. Multivariate logistic regression analysis was done to identify the risk factors of the prognosis of patients with acute attack of CHF acute attack. ROC curves were plotted to evaluate the predictive value of galectin-3, RDW, Hepc, HS and ferritin levels at admission for the prognosis of patients with acute attack of CHF. The test level was set at α = 0.05.

## Results

### Comparison of clinical data between the two groups

The effectiveness rate was 71.74% (66/92) for 92 patients with acute attack of CHF and the non-effectiveness rate was 28.26% (26/92). There was no significant difference in sex, age, NYHA cardiac function class, comorbidities, history of smoking and drinking between the two groups (*P* > 0.05), as shown in Table 1. However, coronary heart disease (CHD), hypertension and valvular heart disease in the effective group were lower than those in the non-effective group, with significant differences (*P* < 0.05).

**Table 1:** Comparison of clinical data between the two groups

Variables	The effective group (n = 66)	The non-effective group (n = 26)	χ^2^	*P*
Sex (male/female) (n)	31/35	14/12	0.353	0.552
Age (years)	59.28 ± 10.65	59.67 ± 10.42	− 0.159	0.874
BMI (kg/m^2^)	23.43 ± 4.48	23.13 ± 4.93	0.375	0.709
NYHA cardiac function class (%)				
II	17 (25.76%)	6 (23.08%)	0.132	0.936
III	19 (28.79%)	7 (26.92%)		
IV	30 (45.46%)	13 (50.00%)		
Comorbidities (%)				
Obesity	6 (9.09%)	4 (15.38%)	0.763	0.383
COPD history	7 (10.61%)	2 (7.69%)	0.179	0.672
Hyperlipidemia	11 (16.67%)	9 (34.62%)	3.532	0.060
Diabetes	14 (21.21%)	5 (19.23%)	0.045	0.833
Hypertension	3(4.55%)	8 (30.77%)	12.185	< 0.001
Drinking (n)	12 (18.19%)	5 (19.23%)	0.014	0.907
Smoking (n)	15 (22.73%)	7 (26.92%)	0.180	0.671
CHD (n)	3 (4.55%)	13 (50%)	26.824	< 0.001
Atrial fibrillation (n)	4 (6.06%)	2 (7.69%)	0.081	0.775
Cerebrovascular disease (n)	9 (13.64%)	4 (15.38%)	0.047	0.828
Valvular heart disease (n)	3 (4.55%)	14 (53.85%)	8.169	0.004
Routine inspection indicators				
BNP before treatment (ng/L)	441.29 ± 78.93	440.78 ± 79.38	0.028	0.978
BNP after treatment (ng/L)	303.29 ± 71.29	337.92 ± 76.28	− 1.701	0.092
NT-pro-BNP before treatment (ng/L)	1461.89 ± 432.09	1459.87 ± 442.97	0.021	0.984
NT-pro-BNP after treatment (ng/L)	518.75 ± 127.98	571.76 ± 136.93	− 1.754	0.083
CTn-I before treatment (ng/L)	245.98 ± 47.98	253.09 ± 49.82	− 0.633	0.528
CTn-I after treatment (ng/L)	193.98 ± 43.29	207.92 ± 47.93	− 1.349	0.181
Serum iron (pg/dI)	19.08 ± 3.29	18.28 ± 3.28	1.051	0.296
Serum sodium (mmol/L)	134.78 ± 30.92	133.87 ± 32.56	0.125	0.903
Serum potassium (mmol/L)	4.21 ± 0.76	4.19 ± 0.72	0.115	0.909
Blood glucose (mmol/L)	5.01 ± 1.05	5.13 ± 1.09	− 0.488	0.627
Glycated hemoglobin (%)	5.37 ± 1.02	5.43 ± 1.05	− 0.207	0.837
Blood urea nitrogen (mmol/L)	6.03 ± 1.73	5.89 ± 1.83	0.335	0.739
Serum creatinine (μmol/L)	93.29 ± 17.28	91.38 ± 16.53	0.493	0.624
eGFR (mL/min/1.73 m^2^)	76.93 ± 12.38	74.73 ± 13.29	0.752	0.454
Echocardiography before treatment				
LVEDd (mm)	68.73 ± 7.09	69.21 ± 7.46	− 0.288	0.774
LVIDs (mm)	43.87 ± 6.87	44.91 ± 6.54	− 0.662	0.502
LVEF (%)	35.58 ± 5.91	34.98 ± 5.03	0.456	0.649
LVPWT (mm)	14.23 ± 3.11	14.45 ± 3.18	− 0.304	0.762
LVST (mm)	14.87 ± 3.21	14.94 ± 3.42	− 0.092	0.927
RA (mm)	62.19 ± 12.87	62.79 ± 13.17	− 0.198	0.842
RVD (mm)	21.76 ± 4.98	21.85 ± 5.65	− 0.075	0.944
Echocardiography after treatment				
LVIDd	49.87 ± 13.26	50.32 ± 13.18	− 0.147	0.883
LVIDs (mm)	39.65 ± 6.59	40.98 ± 6.75	− 0.866	0.389
LVEF (%)	47.93 ± 5.37	46.09 ± 5.48	1.404	0.640
LVPWT (mm)	10.37 ± 2.78	11.09 ± 2.87	− 1.108	0.271
LVST (mm)	10.32 ± 3.01	10.89 ± 2.98	− 0.819	0.414
RA (mm)	49.38 ± 10.28	50.38 ± 10.03	− 0.423	0.673
RVD (mm)	16.03 ± 3.45	16.34 ± 3.56	− 0.385	0.701

NYHA, New York Heart Association; COPD, chronic obstructive pulmonary disease; CHD, coronary heart disease; BNP, B-type natriuretic peptide; NT-pro-BNP, N-terminal pro-BNP; cTn, cardiac troponin; eGFR, estimated glomerular filtration rate; LVEDd, left ventricular end-diastolic diameter; LVIDs, left ventricular internal dimension systole; LVEF, left ventricular ejection fraction; LVPWT, left ventricular posterior wall thickness; LVST, left ventricular septal thickness; RA, right atrium; RVD, right ventricle diameter

### Comparison of the levels of galectin-3, RDW, Hepc, HS and ferritin before and after treatment between the two groups

Before treatment, the effective group had lower levels of galectin-3, RDW, Hepc, and HS than the non-effective group and had higher ferritin levels than the non-effective group, and the difference was significant (*P* < 0.05). After treatment, the effective group had lower levels of galectin-3, RDW, Hepc, and HS than the non-effective group and had higher ferritin levels than the non-effective group, and the difference was significant (*P* < 0.05), as shown in Table 2. In addition, the follow-up results of the two groups showed that the rate of cardiac death and readmission in the effective group was lower than that in the non-effective group, and the difference was statistically significant (*P* < 0.05). There was no significant difference in malignant arrhythmia and acute coronary syndrome between the two groups (*P* > 0.05), seen in Table 3.

**Table 2:** Comparison of the levels of galectin-3, RDW, Hepc, HS and ferritin before and after treatment between the two groups

Variables	Time	The effective group (n = 66)	The non-effective group (n = 26)	t	*P*
Galectin-3 (n)	Before treatment	25.39 ± 3.21	31.29 ± 3.12	− 8.000	< 0.001
	After treatment	15.27 ± 3.03	19.27 ± 3.09	− 5.670	< 0.001
RDW (fL)	Before treatment	45.39 ± 3.21	48.48 ± 3.09	− 4.200	< 0.001
	After treatment	41.28 ± 3.12	44.73 ± 3.02	− 4.818	< 0.001
Hepc (ng/mL)	Before treatment	93.02 ± 9.87	102.98 ± 9.12	− 4.449	< 0.001
	After treatment	57.27 ± 9.12	66.35 ± 9.01	− 4.314	< 0.001
HS (μg/L)	Before treatment	191.98 ± 8.32	199.39 ± 8.28	− 3.852	< 0.001
	After treatment	179.28 ± 7.66	185.31 ± 7.83	− 3.379	0.001
Ferritin (ng/mL)	Before treatment	28.93 ± 12.12	23.02 ± 11.87	2.118	0.037
	After treatment	117.98 ± 13.02	109.98 ± 12.98	2.656	0.009

**Table 3:** Comparison of follow-up results between the two groups, % (n)

Variables	The effective group (n = 66)	The non-effective group (n = 26)	χ^2^	*P*
Cardiac death	0	2 (7.69%)	5.189	0.023
Malignant arrhythmia	1 (1.52%)	2 (7.69%)	2.256	0.133
Acute coronary syndrome	1 (1.52%)	2 (7.69%)	2.256	0.133
Readmission rate	3 (4.55%)	5 (19.23%)	5.066	0.024

### Correlation analysis of the prognosis of patients with acute attack of CHF and galectin-3, RDW, Hepc, HS and ferritin

Spearman correlation analysis showed that the effectiveness of treatment of patients with acute attack of CHF was positively correlated with galectin-3, RDW, Hepc, and HS (r = 0.217, 0.109, 0.376, 0.765, *P* = 0.026, 0.032, 0.021, 0.006), and negatively correlated with ferritin (r = − 0.127, *P* = 0.037).

### Univariate logistic regression analysis of risk factors of the prognosis of patients with acute attack of CHF

Univariate logistic regression analysis was done using hypertension, CHD, valvular heart disease, galectin-3, RDW, Hepc, HS and ferritin as independent variables, and the effectiveness of treatment of patients with acute attack of CHF as a dependent variable. The results showed that hypertension, CHD, valvular heart disease, galectin-3, RDW, Hepc, and HS were adverse predictors and ferritin was a favorable predictor of the prognosis of patients with acute attack of CHF, as shown in Table 4.

**Table 4:** Univariate logistic regression analysis of risk factors of acute myocardial infarction concomitant with heart failure

Variables	β	SE	Wdlodχ^2^	OR (95%CI)	*P*
Hypertension	0.367	0.154	5.107	1.412 (1.041–1.786)	0.021
CHD	0.153	0.039	15.298	1.158 (1.048–1.256)	< 0.001
Valvular heart disease	0.357	0.147	5.108	1.421 (1.037–1.978)	0.021
Galectin-3	0.608	0.187	9.751	1.817 (1.117, 2.376)	0.003
RDW	0.329	0.107	8.676	1.367 (1.198, 1.587)	< 0.001
Hepc	0.647	0.212	10.047	1.876 (1.165, 2.965)	< 0.001
HS	0.356	0.147	5.113	1.401 (1.037, 1.953)	0.019
Ferritin	− 0.408	0.127	10.001	0.612 (0.378, 0.979)	< 0.001

CHD, Coronary heart disease

### Multivariate logistic regression analysis of risk factors of the prognosis of patients with acute attack of CHF

Multivariate logistic regression analysis was done using hypertension, CHD, valvular heart disease, galectin-3, RDW, Hepc, HS and ferritin as independent variables, and the effectiveness of treatment of patients with acute attack of CHF as a dependent variable. The results showed that hypertension, CHD, valvular heart disease, alectin-3, RDW and, Hepc were adverse predictors of the prognosis of patients with acute attack of CHF, as shown in Table 5. Additionally, the optimal threshold points of galectin-3, RDW, Hepc, HS and ferritin for predicting the prognosis of patients with acute exacerbation of chronic heart failure were 31.287 ng/ml, 48.769 fL, 102.969 ng/mL, 199.389 μg/L, 23.022 ng/mL, respectively.

**Table 5:** Multivariate logistic regression analysis of risk factors of acute myocardial infarction concomitant with heart failure

Variables	β	SE	Wdlodχ^2^	OR (95%CI)	*P*
Hypertension	0.007	0.003	4.837	1.006 (0.087–1.074)	0.023
CHD	0.079	0.042	5.893	1.083 (1.009–1.182)	0.012
Valvular heart disease	0.059	0.025	4.562	1.091 (1.003–1.112)	0.029
Galectin-3	1.708	0.687	11.341	3.017 (1.117, 5.376)	< 0.001
RDW	0.719	0.213	10.892	1.987 (1.298, 3.587)	< 0.001
Hepc	3.329	0.839	17.278	1.876 (1.165, 2.965)	< 0.001
HS	0.413	0.219	3.279	1.504 (0.727, 4.467)	0.092
Ferritin	− 0.026	0.069	0.187	0.309 (0.211, 1.393)	0.119

CHD, Coronary heart disease

## Discussion

CHF is a common illness in cardiovascular medicine department and is the terminal state of multiple cardiovascular diseases. Patients with acute attack of CHF have hepatic and gastrointestinal tract hemorrhage and edema of the subcutaneous tissues due to cardiac volume overload and body fluid retention [10]. The main clinical manifestations are dyspnea, fatigue and tissue edema and most patients have a poor prognosis. Therefore, it is important clinically to identify effective prognostic predictors which could facilitate designing treatment plan and making medical decisions in the early stage and help improve the prognosis of patients.

Hypertension-related heart failure initially manifests as asymptomatic left ventricular diastolic dysfunction, followed by left ventricular hypertrophy, which then develops into heart failure [11]. Therefore, it is a risk factor for hypertensive heart failure, and heart failure is also the most important and most serious complication of hypertension. CHF is a complex clinical syndrome caused by abnormal cardiac structure and function, which is the terminal severe stage of CHD and the main cause of death [12]. Previous studies have pointed out that CHF is a risk factor for CHD, and valvular heart disease can cause structural changes and dysfunctions of the atrium and ventricle, and eventually lead to heart failure and arrhythmia. Therefore, valvular heart disease is an important risk factor of heart failure [13, 14].

Most recent studies have shown that iron deficiency is closely related with the severity of CHF, and is considered to be an effective predictor of the outcome of CHF [15]. Hepc is a peptide hormone and a major regulator of iron contents in the body. It interacts with transferrin on the epithelial cells of the intestine, macrophage and the membrane of hepatocytes, restricting the entry of iron into the systemic circulation [16]. Hepc is downregulated when iron is deficient to promote iron absorption and increase iron supply to prevent pathological iron excess. The study by Fu et al. [16] showed that Hepc and ferritin were intimately associated with the severity of CHF. Serum ferritin is an omnipresent protein and an important component of myocardial energy metabolism and structural proteins [17]. Currently, serum ferritin content is used to determine whether iron deficiency is present. The study by Xing et al. [17] revealed that serum ferritin levels decreased with decline in cardiac function of CHF patients with anemia and helped determine the severity of cardiac function of HF patients with anemia and could provide a reference for prediction of changes of disease in the patients. Heart failure is characterized by extensive inflammation. Meanwhile, galectin-3 is an inflammatory mediator and can recruit macrophage and fibroblasts locally and promote the degradation and deposition of collagen [18]. The study by Jia et al. [18] demonstrated that CHF patients had markedly increased serum galectin-3 levels which were closely related to the severity of disease in patients. Clinically, determination of serum galectin-3 levels could provide reference for diagnosis and assessment of prognosis of CHF. RDW is a laboratory parameter reflecting the heterogeneity of erythrocytes and increase in RDW is likely associated with heart failure, stroke, colon cancer, inflammatory bowel disease, pregnancy and pulmonary hypertension [19]. Recent studies have shown that RDW is a novel marker for predicting the prognosis of heart failure patients prognosis [19]. The study by Zhang et al. [20] showed that RDW was intimately related to the cardiac function of CHF patients. Chemokines play an important role in inflammatory reaction and could regulate energy metabolism equilibrium, promote neoangiogenesis and are involved in the proliferation of endothelial cells [21]. The study by Wang et al*.* [22] demonstrated that serum chemokine levels were increased in CHF patients, but decline after effective treatment. The current study showed that before treatment, the effective group had lower levels of hypertension, CHD, valvular heart disease, galectin-3, RDW, Hepc, and HS and higher levels of ferritin than the non-effective group (*P* < 0.05). After treatment, the effective group had lower levels of galectin-3, RDW, Hepc, and HS and higher ferritin levels than the non-effective group (*P* < 0.05), suggesting that galectin-3, RDW, Hepc, HS and ferritin are expressed in high levels in patients with acute attack of CHF, and in even higher levels in patients with a poor prognosis. After effective treatment, their levels showed a tendency of decline. The incidence of hypertension, CHD, and valvular heart disease was higher in patients with poor prognosis in patients with acute exacerbation of CHF.

Previous studies on galectin-3, RDW, Hepc, HS and CHF mainly focused on cardiac function. For example, the study by Guo et al*.* [23] showed that serum ferritin was closely associated with the cardiac function of CHF patients and can be used as an indicator of cardiac function. The study by Xu et al*.* [24] demonstrated that serum galectin-3 levels increased in CHF patients, and NYHA cardiac function class was closely related to cardiac function. The study by Shi et al*.* [25] revealed that increased RDW was correlated with worsening cardiac function in elderly CHF patients. Another study [12] showed that Hepc levels were increased in CHF patients and were notably higher than those in normal persons and were positively correlated with left ventricular mass index and can be used to assess the severity of CHF. However, very few studies are available on galectin-3, RDW, Hepc, HS and the prognosis of patients with acute attack of CHF. In the current study, Spearman correlation analysis revealed that galectin-3, RDW, Hepc, and HS were positively correlated with the prognosis of patients with acute attack of CHF (*P* = 0.026, 0.032, 0.021, 0.006), and ferritin was negatively correlated with the prognosis of patients with acute attack of CHF (*P* = 0.037), indicating that galectin-3, RDW, Hepc, HS and ferritin are closely associated with the prognosis of patients with acute attack of CHF.

Di [12] and other studies pointed out that hypertension is a risk factor for CHF. Bechthold [26] and other studies pointed out that the risk factors of CHF include CHD. Research by Liu [27] et al. pointed out that valvular heart disease is a risk factor for CHF. The study by Merle et al*.* [28] showed that elderly CHF patients without anemia also had iron deficiency and reduced levels of HS and ferritin. The study by Huang et al*.* [29] demonstrated that serum galectin-3 was highly expressed in CHF and was correlated with the prognosis of CHF. The study by Wang et al. [30] revealed that RDW levels were closely correlated with the prognosis of patients with acute heart failure, and effectively predict the 1-year mortality rate. The study by Ma et al. [31]等showed that serum chemokines were abnormally elevated in CHF patients and could be used as effective biomarkers for the evaluation of CHF. In the current study, univariate logistic regression analysis showed that galectin-3, RDW, Hepc, and HS were adverse predictors of the prognosis of patients with acute attack of CHF while ferritin was a favorable predictor of the prognosis of patients with acute attack of CHF. The study further demonstrated that galectin-3, RDW, and Hepc were adverse predictors of the prognosis of patients with acute attack of CHF. Possible mechanisms are as follows: (1) galectin-3 could induce the migration of macrophage to the cardiac tissues and activate fibroblast, facilitating myocardial fibrosis [29]. (2) When RDW level increases beyond 14%, the deformability of red blood cells in the capillary is reduced, affecting blood flow in the capillary, and leading to hypoxia in the microcirculation and ultimately heart failure. Furthermore, increased RDW levels increase red blood cells and aggravate hypoxia, triggering heart failure. Inflammation suppresses the maturation of red blood cells and accelerates the migration of reticulocytes to the peripheral blood, promoting the rise in RDW levels [30]. (3) chemokines cause vasoconstriction and promote release of proinflammatory cytokines by inhibiting adenosine cyclase activities, aggravating inflammatory reaction. HS on the surface of endothelial cells can interact with multiple chemokines and selectins on the surface of leucocytes, promoting inflammatory reaction, which may increase respiratory infection in CHF patients. When CHF patients have respiratory infection, massive amounts of pathogenic bacteria release toxins, leading to myocardial ischemia and compromising cardiac function. Meanwhile, activation of the sympathetic nerves causes constriction of peripheral blood vessels and increases cardiac load, leading to acute aggravation of CHF [31].

However, the study has the following limitations: (1) this is a single center study with a limited sample size and there is the risk of selection bias; (2) galectin-3, RDW, Hepc, HS and ferritin levels were evaluated only before and after treatment and were not dynamically assessed during treatment; (3) This study was a retrospective analysis and did not prospectively study the impact on patient outcomes after long-term follow-up, which may lead to certain limitations in the interpretation of the current results. Therefore, further studies are required, especially multicenter prospective studies with a greater sample size.

In summary, galectin-3, RDW, Hepc, HS and ferritin are highly expressed in patients with acute attack of CHF, and higher levels of them were related to the poorer prognosis. In addition, galectin-3, RDW, and Hepc were risk factors for the prognosis of patients with acute attack of CHF. The results may provide some data support for the future clinical application of galectin-3, RDW, and Hepc to predict the prognosis of acute attack of CHF, and for early prevention and treatment of acute attack of CHF.

## Data Availability

All data generated or analysed during this study are included in this published article.

## Abbreviations

CHF: Chronic heart failure
CHD: Coronary heart disease
Hepc: Hepcidin
HS: Heparan sulfate
